# TREX2 deficiency suppresses spontaneous and genotoxin-associated mutagenesis

**DOI:** 10.1016/j.celrep.2023.113637

**Published:** 2024-01-03

**Authors:** Teresa Marple, Mi Young Son, Xiaodong Cheng, Jun Ho Ko, Patrick Sung, Paul Hasty

**Affiliations:** 1 Department of Molecular Medicine and Institute of Biotechnology, University of Texas Health San Antonio, San Antonio, TX 78229, USA; 2 Department of Biochemistry and Structural Biology, University of Texas Health San Antonio, San Antonio, TX 78229, USA; 3 The Mays Cancer Center, University of Texas Health San Antonio MD Anderson Cancer Center, San Antonio, TX 78229, USA; 4 Greehey Children’s Cancer Research Institute, University of Texas Health San Antonio, San Antonio, TX 78229, USA; 5 Sam and Ann Barshop Institute for Longevity and Aging Studies, University of Texas Health San Antonio, San Antonio, TX 78229, USA; 6 Present address: Texas Tech University Health Science Center, Midland, TX 79705, USA; 7 Present address: Novartis Gene Therapies, San Diego, CA 92121, USA; 8 These authors contributed equally; 9 Lead contact

## Abstract

TREX2, a 3′–5′ exonuclease, is a part of the DNA damage tolerance (DDT) pathway that stabilizes replication forks (RFs) by ubiquitinating PCNA along with the ubiquitin E3 ligase RAD18 and other DDT factors. Mismatch repair (MMR) corrects DNA polymerase errors, including base mismatches and slippage. Here we demonstrate that TREX2 deletion reduces mutations in cells upon exposure to genotoxins, including those that cause base lesions and DNA polymerase slippage. Importantly, we show that TREX2 generates most of the spontaneous mutations in MMR-mutant cells derived from mice and people. TREX2-induced mutagenesis is dependent on the nuclease and DNA-binding attributes of TREX2. RAD18 deletion also reduces spontaneous mutations in MMR-mutant cells, albeit to a lesser degree. Inactivation of both MMR and TREX2 additively increases RF stalls, while it decreases DNA breaks, consistent with a synthetic phenotype.

## In brief

Mismatch repair (MMR) is a tumor suppressor pathway that suppresses mutations. Marple et al. find that the 3′–5′ exonuclease TREX stabilizes replication forks but also causes spontaneous and genotoxin-stress-induced mutations. In MMR-deleted cells, TREX2 causes nearly all mutations and has an additive defect on replication when co-depleted with MMR.

## Graphical Abstract

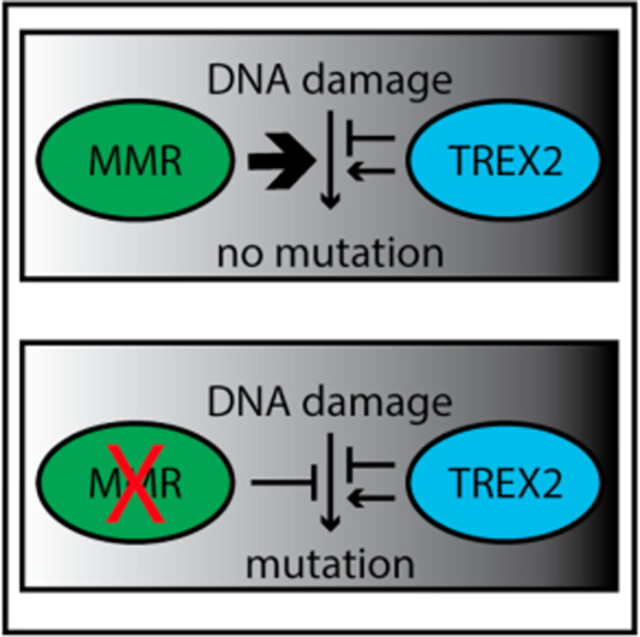

## INTRODUCTION

Mismatch repair (MMR) ensures fidelity of DNA replication by removing mismatches and loops introduced as a result of polymerase errors.^1–3^ The MSH2-MSH6 (MutSα) and MSH2-MSH3 (MutSβ) heterodimers recognize these DNA mismatches and loops. MLH1 forms a complex with PMS2, PMS1, or MLH3 and these complexes bind with MutSα/MutSβ to incise DNA and initiate mismatch or loop removal. A mutator phenotype results when MMR is defective.^4,5^ Inactivation of MMR genes predisposes individuals to Lynch syndrome, a condition that leads to cancer, especially colorectal cancer.^6–9^

DNA damage tolerance (DDT) processes DNA lesions to stabilize replication forks (RFs). There are two mechanisms to handle DNA lesions.^10,11^ In translesion synthesis (TLS), the DNA lesions are bypassed by specialized DNA polymerases, and many of the TLS polymerases are mutagenic.^12,13^ The second mechanism is DNA template switch (TS), which bypasses lesions that could lead to chromosome rearrangements if one strand anneals to a nonallelic repeat on the sister chromatid.^14^ PCNA ubiquitination controls both DDT branches. The RAD6/RAD18 complex is the E2/E3 pair responsible for monoubiquitinating PCNA K164 to induce TLS, while UBC13/MMS2/RAD5 is the E2/E3 responsible for polyubiquitinating the same lysine to induce TS.^11^

TREX2 (Three prime repair exonuclease 2) is a 3′–5′ exonuclease that removes 3′ mismatches from DNA ends.^15–17^ Previously, we demonstrated that TREX2 is a component of DDT because it associates with UBC13 and is essential for optimal PCNA ubiquitination.^14,18^ Importantly, null alleles of TREX2 and RAD18 phenocopy one another. Here we report that TREX2 deletion reduces most genotoxin-induced mutations in wild-type mouse embryonic stem cells (ESCs), and its deletion or depletion reduces spontaneous mutations in MMR-defective ESCs and human-derived cancer cells. RAD18 deletion also reduces spontaneous mutations but to a lesser degree than TREX2 deletion. Furthermore, TREX2 deletion in MMR-mutant cells decreases survival of agents that inhibit DNA replication, increases stalled forks, and decreases DNA breaks, indicating that TREX2 acts in a mutagenic DDT pathway in parallel to MMR.

## RESULTS

### Generation of mouse embryonic stem cells altered for TREX2

All of the *Trex2* coding sequences are located on a single exon, and all of these sequences were deleted in mouse ESCs using our knockout/knockin protocol.^19,20^ The targeting vector used a floxed mouse *Trex2* cDNA, *HPRT* (hypoxanthine phosphoribosyl-transferase) minigene (*miniHPRT*). The minigene offers dual selection in HAT (hypoxanthine, aminopterin, thymidine) for function or in 6-thioguanine (TG) against function.^21,22^ Transfected cells were selected in HAT after transfection (Figure S1A1). Next, a Cre-recombinase plasmid was transfected to remove the 5′ region of *miniHPRT* after TG selection (Figure S1A2). To generate a knockin, a Cre-mediated targeting vector that contains the 5′ half of *miniHPRT* along with wild-type human *TREX2* (TREX2^WT^) cDNA or empty vector (TREX2^null^) was co-transfected with Cre recombinase and selected in HAT (Figure S1A3). *Trex2* is on the X chromosome, so only one copy needs to be deleted in the XY cells used here.

### TREX2 generates spontaneous and genotoxin-induced mutations

**Figure 1.**
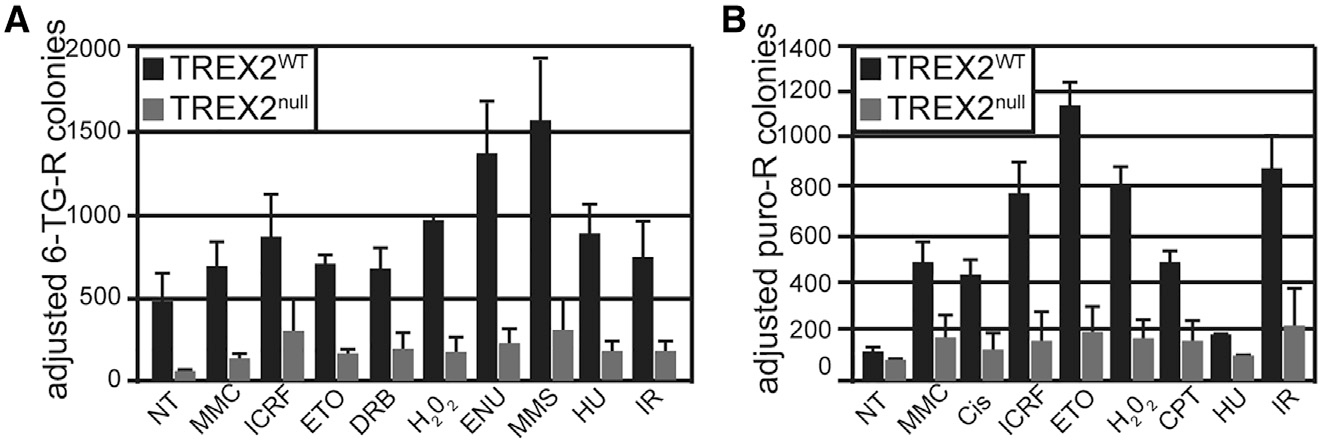
Genotoxin-induced mutations require TREX2 The impact of TREX2 on genotoxin-induced mutations using the (A) *miniHPRT* LOF and (B) *puro(A)*_*10*_ polymerase slippage assays. These results are adjusted to the seeding efficiency control plate (number of colonies that grow without exposure to agent). Statistics for (A) and (B) are the average of three replicates using the unpaired Student’s t test and are listed in the text; mean ± SD; n = 3. ESCs were exposed to the following genotoxins: no treatment (NT), mitomycin C (MMC), camptothecin (CPT), hydroxyurea (HU), hydrogen peroxide (H_2_O_2_), N-ethyl-N-nitrosourea (ENU), methylmethane sulfonate (MMS), cisplatin (Cis), etoposide (ETO), meso-4,4-(2,3-butanediyl)-bis-(2,6-piperazinedione) (ICRF-193), ionizing radiation (IR), and 5,6-dichloro-1-β-D-ribofuranosylbenzimidazole (DRB).

TREX2^WT^ and null cells were exposed to a variety of genotoxins, including DNA cross-linking agents (mitomycin C [MMC] and cisplatin), a type 1 topoisomerase inhibitor (camptothecin [CPT]), type 2 topoisomerase inhibitors/poisons (ICRF-193 and etoposide [ETO]), DNA alkylating agents (N-ethyl-N-nitrosourea [ENU] and methylmethane sulfonate [MMS]), reactive oxygen species (hydrogen peroxide [H_2_O_2_]), a ribonucleotide reductase inhibitor (hydroxyurea [HU]), and ionizing radiation (IR) (Figure 1). In general, DNA cross-linking agents, topoisomerase inhibitors, and IR induce double-strand breaks (DSBs), while reactive oxygen species and alkylating agents cause base lesions.^23^ Cells were also exposed to an RNA polymerase II inhibitor (5,6-dichloro-1-β-D-ribofuranosylbenzimidazole [DRB]) that slows transcription elongation. DRB can cause DNA damage via the induction of Z-DNA formation, a non-B structure of DNA.^24^ These structures can lead to genomic fragile sites that cause DNA breaks and translocations.^25^ The *miniHPRT* loss-of-function (LOF) assay was used to measure mutations by resistance to TG.^18,26^ Without genotoxin treatment, TREX2^WT^ cells displayed significantly more spontaneous mutations compared with TREX2^null^ cells (Figure 1A, p = 0.0074).

Exposure to all genotoxins increased mutations in both genotypes (Figure 1A, TREX2^WT^ cells, p < 0.05; TREX2^null^ cells, p < 0.013), but the level of mutations remained lower for TREX2^null^ cells. These data support the premise that TREX2 is responsible for converting a variety of DNA lesions into genetic mutations spontaneously and upon treatment with a genotoxin.

We used *puro(A)*_*10*_ as a reporter for DNA polymerase slippage. *puro(A)*_*10*_ is the puromycin (puro) acetyltransferase cassette with an out-of-frame insert of 10 adenines immediately downstream of the initiation ATG. To test *puro(A)*_*10*_, cells described in Figure S1C were transfected with a *flippase* recombinase plasmid to remove *miniHPRT* and selected in TG (Figure S1A4). Into these cells the *miniHPRT* plasmid was co-transfected with *puro(A)*_*10*_. HAT-resistant colonies were pooled and then selected in puro to reveal cells that had undergone polymerase slippage to restore the reading frame. Without genotoxin treatment, TREX2^null^ cells accumulated fewer slippage mutations than TREX2^WT^ cells (Figure 1B, p = 0.026). Exposure to all genotoxins significantly increased the number of puro-resistant colonies in TREX2^WT^ cells (Figure 1B, p < 0.05), while TREX2 deletion significantly lowered slippage mutations (Figure 1B, p < 0.0058). We conclude that TREX2 is crucial for the generation of polymerase slippage mutations both spontaneously and in response to a variety of genotoxins.

### TREX2 generates spontaneous polymerase-slippage-induced mutations in MMR-deficient cells

**Figure 2.**
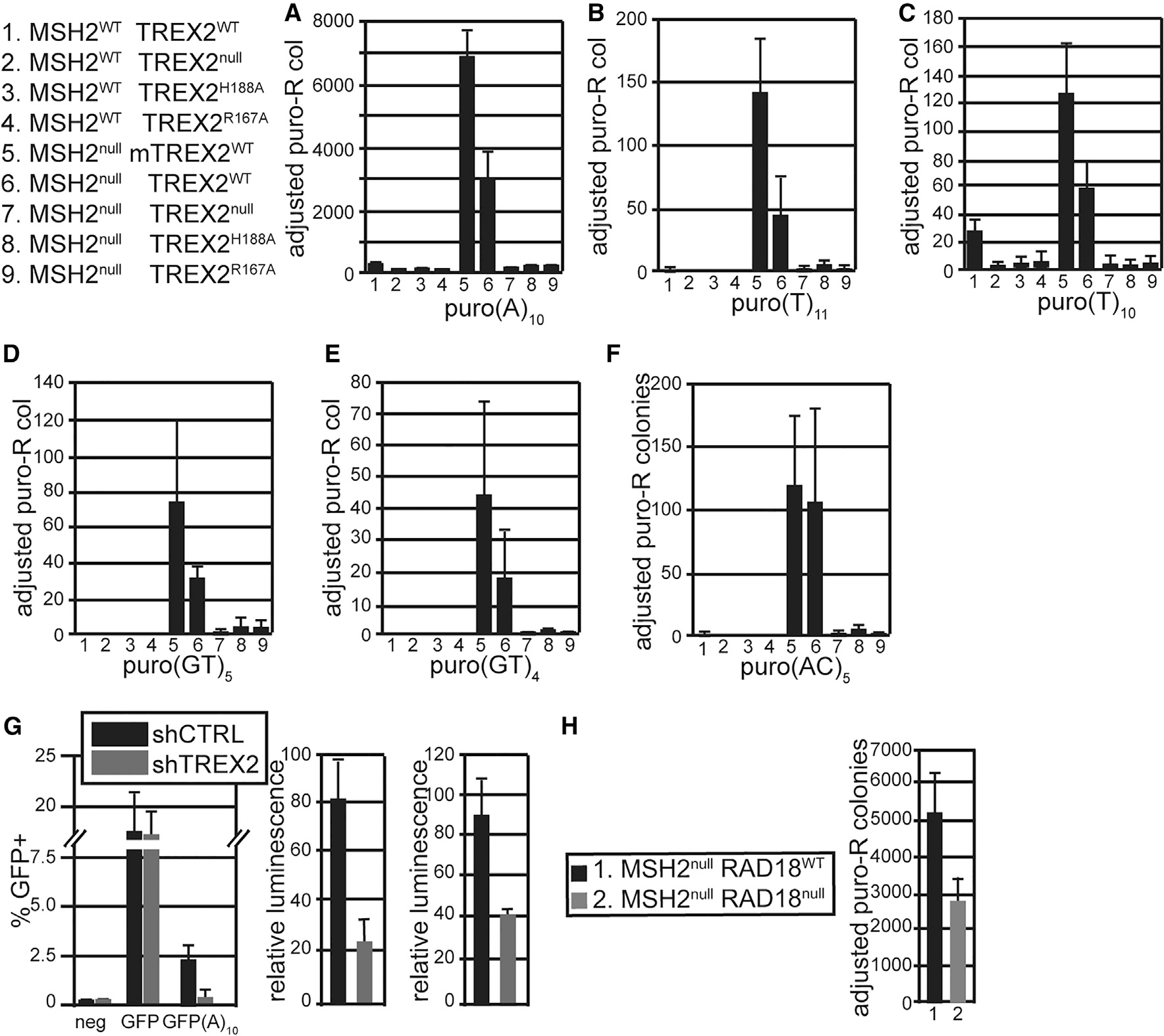
TREX2 caused polymerase slippage-induced mutations in MMR-mutant ESCs and in human-cancer-derived cells (A–F) Polymerase slippage was measured with a puro reporter with the following out-of-frame inserts: (A)_10_, (T)_11_, (T)_10_, (GT)_5_, (GT)_4_, and (AC)_5_. (G) Polymerase slippage was measured in human-derived cancer cells. A *GFP(A)*_*10*_ reporter was transfected into HCT116 cells, derived from human colorectal carcinoma (ATCC CCL-247), shown on the left. A *Luciferase(A)*_*10*_ reporter was transfected into SK-OV-3 cells, derived from ovarian adenocarcinoma (ATCC HTB-77), and into CCRF-CEM (ATCC CCL-199) cells, derived from acute lymphoblastic leukemia, shown in the middle and right, respectively. (H) RAD18 deletion reduced polymerase slippage of *puro(A)*_*10*_. Statistics for (A)–(D) are the average of three replicates using the unpaired Student’s t test and are listed in the text; mean ± SD; n = 3.

MMR corrects polymerase slippage errors^3^; therefore, in cells with and without MSH2,^27,28^ TREX2^WT^ and TREX2^null^ were compared. In addition, mutations that ablate TREX2’s exonuclease activity (H188A) and diminish DNA binding by 80% (R167A) were analyzed.^15^ Compared with MSH2^WT^ TREX2^WT^ cells, TREX2^null^ or expression of TREX2^H188A^ or TREX2^R167A^ did not increase the number of puro-resistant colonies (Figure 2A, compare lane 1 with lanes 2–4). Compared with MSH2^WT^ cells, MSH2^null 27,28^ increased the number of puro-resistant colonies by ~45- to 18-fold when expressing mouse or human TREX2^WT^, respectively (Figure 2A, compare lane 1 with lanes 5 and 6, p < 0.01). Deleting TREX2 in MSH2^null^ cells reduced puro-resistant colonies by 230- to 100-fold (Figure 2A, compare lanes 5 and 6 with lane 7, p < 0.0001). In addition, expressing TREX2^H188A^ or TREX2^R167A^ reduced the number of puro-resistant colonies by ~100- to75-fold, respectively (Figure 2A, compare lanes 5 and 6 with lanes 8 and 9, p < 0.0001). Polymerase slippage was further analyzed with a variety of out-of-frame repetitive inserts in puro, including (T)_11_, (T)_10_, (GT)_5_, (GT)_4_, and (AC)_5_. MSH2 deletion increased puro-resistant colonies for all these inserts, and TREX2 deletion or expression of TREX2^H188A^ or TREX2^R167A^ reduced the number of mutations (Figures 2B–2F, p < 0.01). These data indicate that TREX2’s exonuclease activity and its DNA-binding function are both required for the generation of polymerase slippage errors in MSH2^null^ cells.

TREX2 causes mutations in human-derived cancer cells mutated for *MLH1*. TREX2 was depleted with short hairpin RNA (shRNA) in HCT116 cells derived from colorectal carcinoma, SK-OV-3 cells derived from ovarian adenocarcinoma, and CCRF-CEM cells derived from lymphoblastic leukemia (Figure S1B). Two frameshift reporters were used to measure polymerase slippage: *GFP(A)*_*10*_ and *Luciferase(A)*_*10*_. Using *GFP(A)*_*10*_, depletion of *TREX2* reduced mutations in HCT116 cells (Figure 2G, left, p = 0.0043). Using *Luciferase(A)*_*10*_, depletion of *TREX2* also reduced mutations in SK-OV-3 cells (Figure 2G, middle, p = 0.0091) and CCRF-CEM cells (Figure 2G, right, p = 0.0155). Together, our results demonstrate that TREX2 generates mutations in human-derived cancer cells as well as in mouse ESCs that are defective in MMR.

### RAD18 generates spontaneous polymerase-slippage-induced mutations in MMR-deficient cells

We wanted to test if a component of DDT, RAD18, participates in mutation induction in MMR-deficient cells.^14,18^ RAD18 was deleted by co-transfecting gRNA/Cas9 and a neomycin selection cassette into MSH2^null^ cells.^29,30^ G418-resistant colonies were isolated to extract protein and genomic DNA for western blot and PCR analysis. A clone was chosen that did not contain protein and showed a reduced PCR fragment size (Figure S1C). These cells were tested for polymerase-slippage-mediated mutations with *puro(A)*_*10*_. Deleting RAD18 in MSH2^null^ cells reduced puro-resistant colonies by nearly half (Figure 2H, p < 0.05). Thus, RAD18 and TREX2 are both responsible for generating polymerase-slippage-induced mutations in MMR-deficient cells, but TREX2 might have a more prominent role, since its depletion reduced puro-resistant colonies by ~100-fold (Figure 2A).

### TREX2 generates spontaneous mutations in *miniHPRT* in MMR-deficient cells

**Figure 3.**
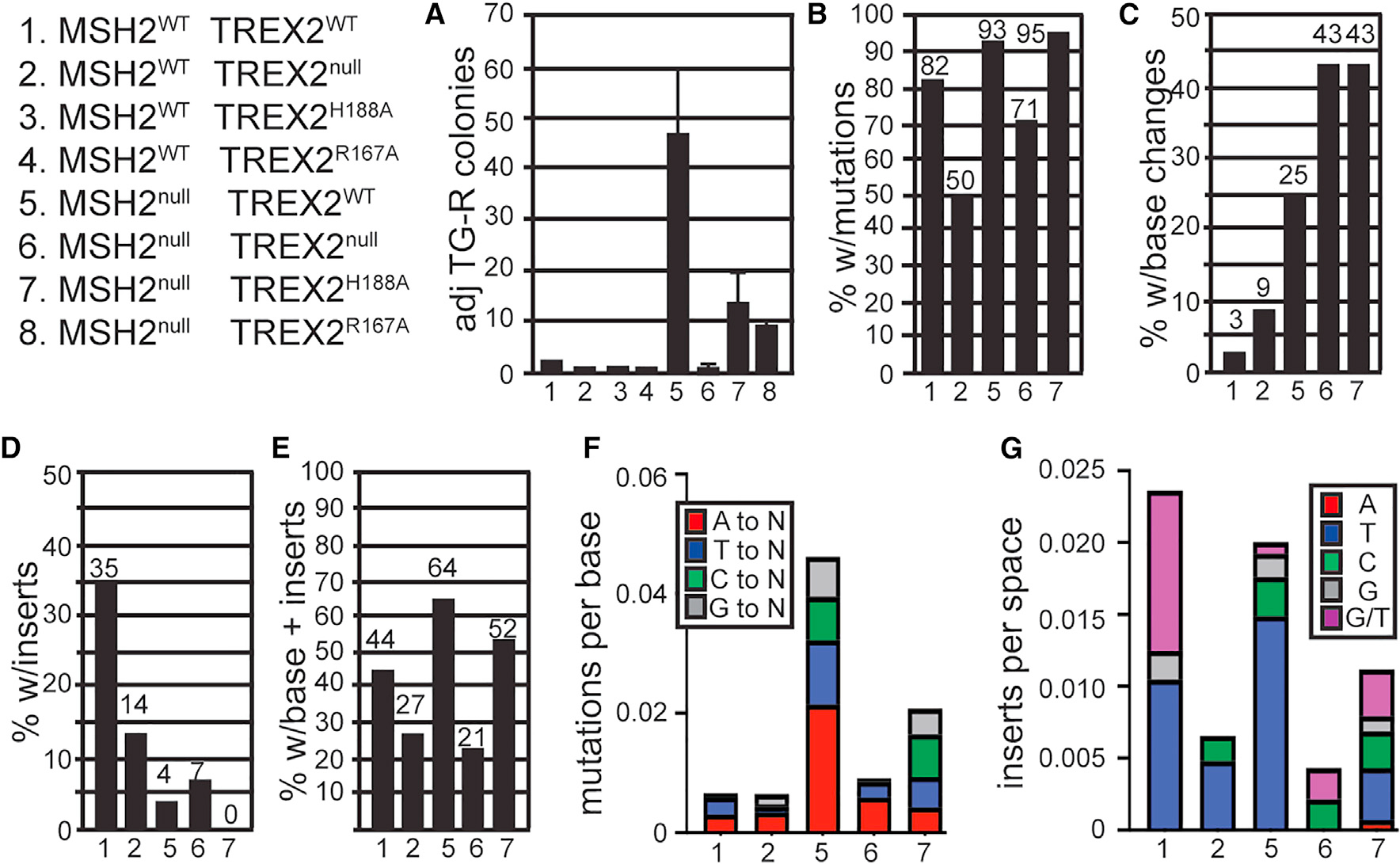
The impact of TREX2 on spontaneous *miniHPRT* LOF (A) Graph showing that TREX2 caused mutations in MSH2^null^ cells. (B) The percentage of TG-resistant clones with a mutation identified by sequence analysis. (C) The percentage of mutant clones that contained only base changes. (D) The percentage of mutant clones that contained only insertions. (E) The percentage of mutant clones that contained both base changes and insertions. (F and G) The mutation rate per base (133) (F) or per space (132) (G) for base changes and inserts, respectively. Statistics for (A)–(D) are the average of three replicates using the unpaired Student’s t test and are listed in the text; mean ± SD; n = 3.

MMR corrects base-base lesions while HPRT causes base lesions in the presence of TG. HPRT is part of the purine salvage pathway that metabolizes TG into thioguanosine monophosphate that is then converted by thiopurine methyltransferase into methylthioguanosine monophosphate, a toxic base analogue that can be incorporated into DNA.^31^ Furthermore, TREX2 caused mutations in cells exposed to base lesion-inducing agents^2^; therefore, the impact of TREX2 deletion was tested on mutation generation in MMR-defective cells using the *miniHPRT* LOF assay. TREX2^null^ or expression of TREX2^H188A^ or TREX2^R167A^ did not increase LOF in cells with functional MMR (Figure 3A, compare lane 1 with lanes 2–4). MSH2 deletion increased the number of TG-resistant colonies by ~30-fold when expressing TREX2^WT^ (Figure 3A, compare lane 1 with lane 5, p < 0.0001). However, in MSH2^null^ cells, deleting TREX2 reduced TG-resistant colonies (Figure 3A, compare lane 5 with lane 6, p < 0.0001) to a level that is approximately the same as control (Figure 3A, compare lane 1 with lane 6). Moreover, expression of either TREX2^H188A^ or TREX2^R167A^ in MSH2^null^ cells reduced mutations compared with TREX2^WT^ expression (Figure 3A, compare lane 5 with lanes 7 and 8, p < 0.001). These data show that TREX2 caused most of the spontaneous mutations in MSH2^null^ cells and that this pro-mutagenic effect of TREX2 is dependent on its nuclease and DNA-binding attributes.

From genomic DNA isolated from TG-resistant clones, we sequenced *miniHPRT* exons 1 and 2 (133 bases from translation-initiating ATG to splice donor [SD]). Mutations were seen in TG-resistant clones for all genotypes (Figure 3B) Base changes and inserts (Figures 3C–3E), but not deletions, were observed.

Base changes were observed. For *Msh2*^+/+^ *Trex2*^*WT*^, there were 0.912 base changes per 133 bp (0.00686 changes per base) (Figure 3F), and deleting TREX2 from these cells had no significant impact (0.864 base changes per 133 bp, 0.0065 changes per base) (Figure 3F). Deleting MSH2 increased the number of base changes by 6.5-fold to 5.93 base changes per 133 bp (0.0446 changes per base) (Figure 3F, compare lane 1 with lane 5, p < 0.0001), and, importantly, deleting TREX2 (Figure 3F, compare lane 5 with lane 6, p < 0.0001) or expressing TREX2^H188A^ (Figure 3F, compare lane 5 with lane 7, p < 0.0001) in these cells decreased the base change rate by 4- and 2.1-fold to 1.5 base changes per 133 bp (0.0113 changes per base) and 2.762 base changes per 133 bp (0.0208 changes per base), respectively. Therefore, TREX2 caused base changes in MMR-deleted cells.

The sequence of base changes was observed (Figure 3F). Adenine was the most common base that was changed when MSH2 was deleted in cells, followed by T and then C and G (p < 0.0001). Deleting TREX2 reduced an adenine change back to the levels that were insignificant (compare lane 5 with lane 6, p = 0.0001, and lane 1 with lane 5), and the same held true for T, C, and G changes (compare lane 5 with lane 6, p < 0.003, and lane 1 with lane 5). Expressing TREX2^H188A^ reduced an adenine change back to the levels that were insignificant (compare lane 5 with lane 7, p = 0.0001, and lane 1 with lane 7), and the same held true for T changes (compare lane 5 with lane 7, p = 0.0179, and lane 1 with lane 7), but not for C and G changes (compare lane 5 with lane 7 and lane 1 with lane 7, p < 0.0019), implying that exonuclease activity was used to remove a subset of base changes. Please refer to Table S1 for the sequences observed.

Inserts were observed. For *MSH2*^+/+^ *Trex2*^*WT*^, there were 3.12 inserts per space (0.0236 per space) (Figure 3G), and deleting TREX2 from these cells reduced this number by 3.6-fold to 0.909 inserts per 132 spaces (0.0069 per space) (Figure 3G, compare lane 1 with lane 2, p < 0.0001), while deleting MSH2 had no significant impact (2.714 inserts per 132 spaces or 0.02 inserts per space) (Figure 3G). For MSH2^null^, deleting TREX2 (Figure 3G, compare lane 5 with lane 6, p < 0.0001) or expressing TREX2^H188A^ (Figure 3G, compare lane 5 with lane 7, p = 0.0083) in these cells decreased the number of inserts by 4.9- and 1.8-fold to 0.571 insert per 132 spaces (0.0043 per space) and 1.476 per 132 spaces (0.0112 per space), respectively. Therefore, TREX2 introduced inserts independent of MSH2.

The sequence of inserts was observed. In wild-type cells, T and G/T inserts were mostly observed (Figure 3G). Compared with wild-type cells, TREX2-deleted cells exhibited decreased levels of T inserts (p = 0.0135) and G/T inserts (p < 0.0001), while MSH2-deleted cells exhibited a decrease in G/T inserts (p < 0.0001) and a small increase in T inserts (p = 0.0956). MSH2-deleted cells deleted for TREX2 or expressing TREX2^H188A^ exhibited decreased levels of T (p < 0.0001 and p = 0.0022, respectively) and G/T inserts (p < 0.0001 and p = 0.0005, respectively) compared with wild type. Please refer to Table S1 for the sequences observed.

### Deletion of MMR and TREX2 leads to an additive RF maintenance defect

TREX2 deletion altered survival of MSH2^null^ cells exposed to genotoxins that induce DNA lesions and/or RF blockage. Cells were exposed to HU, aphidicolin (APH), and CPT. HU is a ribonucleotide reductase inhibitor,^32^ and APH is a DNA polymerase α inhibitor^33^; these agents stall RFs.^34–36^ CPT is a type 1 topoisomerase inhibitor that generates DSBs at RFs.^37^ Dose-response curves to HU, APH, and CPT were performed on cells with and without MSH2 and TREX2. MSH2^null^ cells were not hypersensitive to any of these agents. *Trex2*^*null*^ cells were hypersensitive to 90 μM HU (Figure S2A, p = 0.0049). The double-mutant cells exhibited greater sensitivity to HU and APH than either single mutant (Figures S2A and S2B, see figure legend for statistics), but neither the single-mutant cells nor the double-mutant cells were hypersensitive to CPT (Figure S2C).

Since both HU and APH inhibit DNA replication, DNA fiber analysis was performed to determine how MSH2 and TREX2 mutations affect RF dynamics. Cells were cultured in CldU (chlorodeoxyuridine) for 20 min to label the nascent replication strand and then exposed to 0.5 mM HU for 1.5 h to stall RFs and then exposed to IdU (iododeoxyuridine) for 20 min to label post-HU replication, either from restart or from a new origin. RF restart is a green (CldU)-red (IdU) fiber, while a stalled RF is a green fiber, and a new origin is a red fiber (Figure S2D, left). Cells for all genotypes that were not treated with HU exhibited no difference (Figure S2D, middle). For HU-treated cells, compared with wild type, MSH2^null^ and TREX2^null^ cells failed to show a significant difference (Figure S2D, right); yet, double-mutant cells exhibited a significant increase in stalled RFs compared with wild-type (p = 0.0045), MSH2^null^ (p = 0.0002), and TREX2^null^ (p < 0.0159) cells. Therefore, simultaneously deleting MSH2 and TREX2 leads to enhanced RF stalling in cells.

We used metaphase spreads (MPSs) to analyze chromatid and isochromatid breaks on cells that were either not treated or treated with CPT. All genotypes exhibited increased levels of breaks, with the exception of MSH2^null^ TREX2^null^, with MSH2^WT^ TREX2^WT^ (0.0063), MSH2^null^ mTREX2^WT^ (<0.0001), and MSH2^null^ mTREX2^WT^ (0.0148) being significant. Interestingly, MSH2^null^ TREX2^null^ MPSs exhibited a significant decrease in breaks after exposure to CPT (0.0119) (Figure S2E, compare lane NT with lane CPT). We next compared MSH2^null^ TREX2^null^ cells exposed to CPT with the other genotypes for breaks. We found a significant decrease in the number of breaks in all genotypes (Figure S2E, compare CPT lane 6 with CPT lanes 1–5, p < 0.0005). These data, in addition to the dose-response curves (Figures S2A and S2B) and the RF data (Figure S2D), suggest that defects in TREX2 and MMR exhibit a synthetic phenotype that consists of increased sensitivity to replication inhibitors and RF stalls along with decreased levels of the number of DNA breaks.

## DISCUSSION

We have conducted a detailed analysis of TREX2’s role in mutagenesis with and without exposure to genotoxins as gauged with the *miniHPRT* LOF and polymerase-slippage assays. Without exposure to genotoxins, TREX2 deletion significantly reduces mutations, implicating a TREX2-mediated response in controlling basal mutation levels. Genotoxin treatment increases mutations in wild-type cells that TREX2 deletion ameliorates to levels observed for wild-type cells without treatment, suggesting that TREX2 converts base lesions, cross-links, and DSBs into mutations. Our DRB data implicate TREX2 as being germane for chromosomal aberrations at fragile sites. Yet, TREX2^null^ cells, with and without genotoxin exposure, also accumulate mutations, which reveals a TREX2-independent mechanism of mutation induction.

Our study also functionally links TREX2 to MMR. While MSH2 deletion significantly increases spontaneous mutation levels using the *miniHPRT* LOF and polymerase-slippage assays, TREX2 deletion reduces these mutations to levels found in wild-type cells. A similar result was observed with expression of TREX2^H188A^ (nuclease mutant) and with TREX2^R167A^ (DNA-binding mutant). Previously, we documented the impact that TREX2 has on mutations and replication for cells expressing RAD51^K133A^.^18^ RAD51 is the recombinase for homologous recombination, a pathway that repairs DSBs and stabilizes RFs.^38–40^ RAD51 forms a filament on single-stranded DNA at RFs, and the K133A mutation causes chromosomal rearrangements and stalled RFs.^26^ In regard to mutations, results presented here are similar to those presented for cells expressing RAD51^K133A^. Deletion of TREX2 reduces spontaneous mutations to wild-type levels in both MSH2^null^ and RAD51^K133A^ cells. However, with regard to replication, MMR and RAD51^K133A^ behave differently. Deletion of either TREX2 or MMR has little impact on replication, yet deletion of both pathways leads to elevated levels of stalled RFs when cells are subject to replicative stress. However, there are more stalled RFs in RAD51^K133A^ cells under replicative stress, and mutations in TREX2 alleviate this RF phenotype. Together, our findings provide compelling evidence that TREX2 is involved in the processing of stressed RFs and, given the stark contrast between cells lacking TREX2 and simultaneously ablated for MMR or expressing RAD51^K133A^, its phenotypic outcome is dependent on the DNA repair pathway that is deficient.

**Figure 4.**
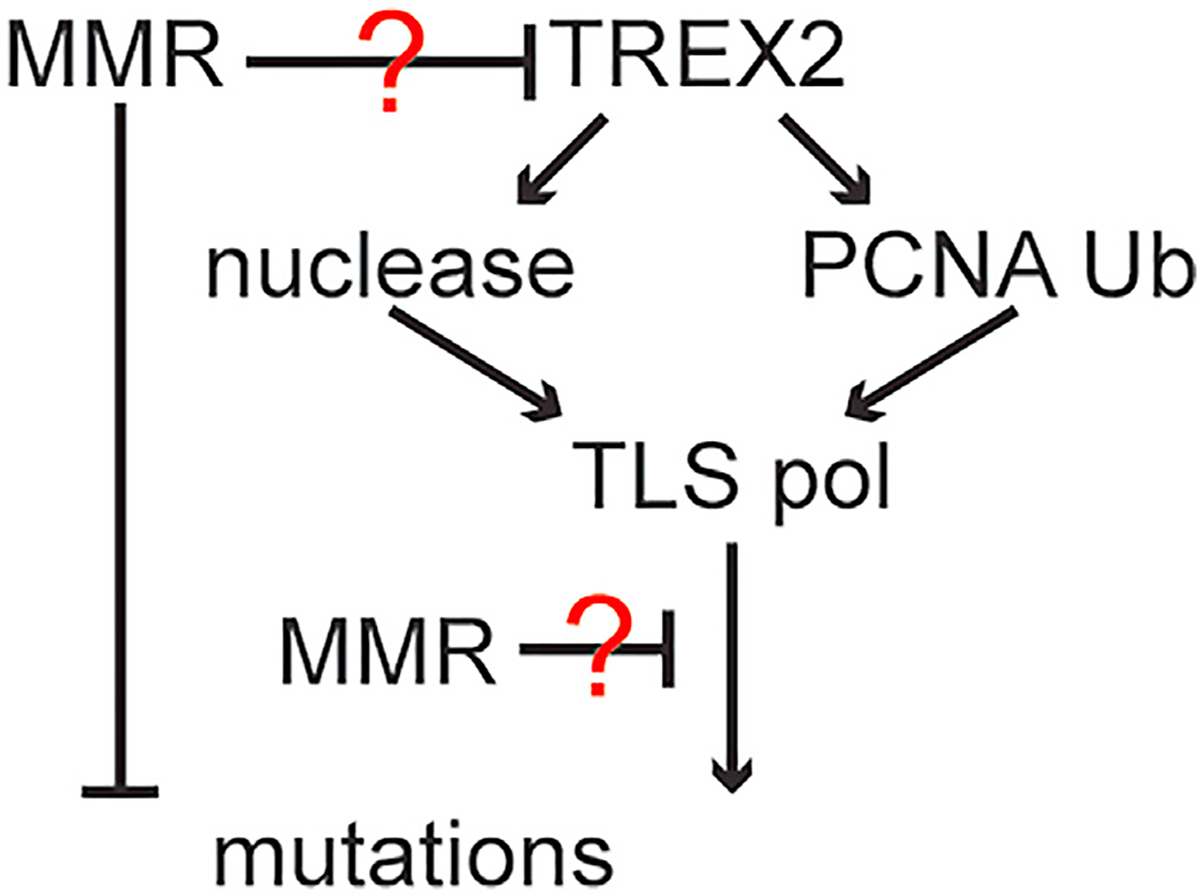
Possible models that illustrate MMR and TREX2 acting at stressed RFs Top ?: MMR is in a pathway parallel to TREX2 and either indirectly or directly suppresses TREX2-mediated TLS-DDT. Bottom ?: TREX2 is upstream of MMR.

We propose several possible models for TREX2-induced mutation generation based on our data that posit that MMR and TREX2 address problems at stressed RFs (Figure 4). MMR suppresses mutations that are caused by mismatches and base lesions, while TREX2 generates mutations that are largely repaired by MMR or caused by MMR deficiency. One possibility is that MMR and TREX2 act in parallel (Figure 4, top question mark), with MMR correcting the mismatch and TREX2, as a part of TLS-DDT, functioning in an alternative pathway.^12,13^ For this model, MMR is dominant to TLS-DDT at stressed RFs. Another possibility is that TREX2 acts first with a mutagenic TLS polymerase to introduce a DNA mismatch that is removed by MMR (Figure 4, bottom question mark). If MMR fails to remove the mismatch, then a mutation can result. We note that TREX2’s exonuclease and PCNA ubiquitination functions are important for causing both spontaneous mutations in an MMR-deficient background. We also note that in addition to correction of mismatches and base lesions, MMR induces DNA-damage-dependent cell-cycle arrest and apoptosis to affect cell proliferation or induces cell death, respectively.^41,42^ As such, MMR could induce a cell-cycle response to the toxic base to stop proliferation or induce death, but in some cases, TREX2 stabilizes stalled RFs to avoid the generation of toxic intermediates.

### Limitations of the study

This study is limited by the scope of mutations that were analyzed. We used two types of reporters: *miniHPRT* and polymerase slippage. The former will detect virtually all mutations but has the caveat of being limited to a single genomic position, while the latter has the advantage of multiple genomic locations but is limited by detection of a single type of defect. We chose to complement this work with both of these reporters, but in the future large-scale sequencing might be better. This study is also limited by the post-replicative pathways that are analyzed. We have studied two of these pathways, MMR and DDT. Others, like replication polymerase proofreading, could be important, since there were still a small number of mutant clones that grew in cells deleted for TREX2.

## STAR★METHODS

Detailed methods are provided in the online version of this paper and include the following:

### RESOURCE AVAILABILITY

#### Lead contact

Correspondence and requests for materials should be addressed to and will be fulfilled by the lead contact, Paul Hasty (hastyhsc@gmail.com)

#### Materials availability

Plasmids and cell lines generated in this study are available without restrictions and will be fulfilled by the lead contact upon request.

#### Data and code availability

- All data in this paper will be available from the lead contact upon request.
- This paper does not report original code.
- Any additional information required to reanalyze the data reported in this paper is available from the lead contact upon request.

### EXPERIMENTAL MODEL AND STUDY PARTICIPANT DETAILS

#### Cell lines

The cell culture conditions for mouse embryonic stem cells (AB1.1 – XY ES cells) have been described^43^. The cells were cultured in Dulbecco’s Modified Eagle’s Medium (DMEM) supplemented with 15% FBS, 2 mM glutamine, 30 μg/mL penicillin, 50 μg/mL streptomycin, 10^−4^ M *β*-mercaptoethanol, and 1000 units/mL 10^7^ mouse leukemia inhibitory factor. The human cancer cell lines HCT116 and SK-OV-3 were cultured in McCoy’s 5A medium supplemented with 10% FBS and CCRF-CEM cells were cultured in RPMI 1640 medium supplemented with 10% FBS. The cells were maintained at 37°C in a 5% CO_2_ humidified incubator.

### METHOD DETAILS

#### Targeting TREX2

The knockout-knockin protocol has been described^19,20^ except the targeting vector used here contained the *MmTrex2* cDNA 3′ to the RE *loxP* and 5’ to *miniHPRT*^19^. Briefly, 5 μg of *MmTrex2* cDNA with *miniHPRT* introduced into mouse AB1.1 ES cells (5 × 10^6^) and the cells were selected with 1x HAT for 8–10 days. The targeted ES cells were transfected with 10 μg of Cre recombinase to delete the 5’ half of *miniHPRT* and they were selected with 10 μM 6-thioguanine (6-TG). Then, the 20 μg of Cre-mediated targeting vector containing human *Trex2* cDNA WT and mutants (null, H188A, R167A) with 5’ half of *miniHPRT* and 10 μg of Cre recombinase introduced to the 6-TG-resistant cells and the cells were selected with 1x HAT to restore *miniHPRT*. To remove the plasmid backbone and *miniHPRT*, the cells were transfected with 5 μg of Flippase recombinase and selected with 10 μM of 6-TG.

#### The *miniHPRT* loss of function (LOF) assay

The miniHPRT LOF assay has been described^18,26^. In brief; mouse ESCs carrying an intact *miniHPRT* cassette (located to Trex2 on the X chromosome after gene targeting) were cultured in medium with 1x HAT (Sigma-Aldrich H0262), trypsinized and passaged every third day, then weaned in medium with 1x HT (Sigma-Aldrich H0137) for 2 days and then transferred to regular medium. Genotoxins were added at this point. After 5 days in regular medium, 1 × 10^5^ cells were transferred to medium containing 10 μM TG (Sigma-Aldrich A4882) for 7–10 days until colonies form. As a seeding control, 1 × 10^4^ cells were seeded in regular medium without 6-TG. Colony numbers were normalized according to colonies on the seeding control plate. ESCs were treated with the following genotoxins from Sigma-Aldrich (unless otherwise stated) for 7 days before adding 6-TG: 10 nM MMC (M4287), 20 nM ICRF-193 (ICN Biomedicals Inc.), 20 nM CPT (C9911), 50 μM HU (H8267), 10 nM ETO (E1383), 100 μM H_2_O_2_ (216763), 5 μM cisplatin (P4394), 100 μM MMS (M4016), 1 mM ENU, 100 μM DRB (Calbiochem 287891). IR dose administered was 1 Gy using a JL Shepherd & Associates MK1-68A Cs-137 Irradiator. Cell viability was >90% by using trypan blue (Gibco 15250-061) exclusion assay for all genotoxins.

#### Sequencing miniHPRT

TG-resistant colonies were picked and expanded. Genomic DNA was extracted and miniHPRT was amplified by PCR using the following custom primers purchased from Sigma-Aldrich.

miniHPRT Exon1-2 Forward: 5’-CTTCAAAAGCGCACGTCTGC-3’, miniHPRT Exon1-2 Reverse: 5’-CAAGTACTCAGAACAGCTGC-3’.

PCR conditions are: 98°C 5 minutes, followed by 40 cycles of reaction (98°C 1 minute, 45°C 1 minute, 72°C 1 minute), final extension is 72°C for 10 minutes. Amplified PCR products were sent for sequence for detection of mutations in *miniHPRT*. The *miniHPRT* Exon1-2 Reverse oligo was used for sequencing.

#### Construction of the polymerase slippage reporter substrates

Construction of *puroRV*: An EcoRV site was cloned immediately downstream of the initiation ATG by using site directed mutagenesis (QuickChange Multi Site-Directed Mutagenesis Kit, Catalog #200514, Stratagene) with the oligo *puroRV*: 5’-ggccaactcaaaggccgccacc ATG GATATC accgagtacaagcccacg-3’ made by the UT Health Science Center San Antonio Nucleic Acids Core facility. *PuroRV* was then cut with EcoRV and complimentary oligos repeats were cloned into this site. The clones were sequenced to orient the oligos.

#### The *puro(A)*_*10*_ polymerase slippage assay

Mouse ESCs were further manipulated to do this experiment. To remove miniHPRT, the cells described in Figure S1C were transfected with a flipase plasmid and were selected in TG as described in Figure S1D. ESCs were cultured in medium until they reach 80% confluence. Medium was changed 2 to 4 hours before the electroporation. ESCs were washed with PBS and digested by trypsin and 5 × 10^6^ cells were re-suspended in 700 uL Ca/Mg free PBS. To this solution, 100 uL PBS containing 5 μg of miniHPRT plasmid and 5 μg *puro(A)*_*10*_ plasmid was added. This mixture was then transferred to 0.4 cm electroporation cuvette and pulsed with 230 V and 500 uF using a BioRad Gene Pulser II (Richmond, CA). The miniHPRT plasmid was transfected to selected for cells that were co-transfected with *puro(A)*_*10*_. Cells were then seeded to 10 cm gelatinized culture dish. After 24 hours, medium was removed and replaced with medium containing 1x HAT. Cells were trypsinized and re-suspended in medium containing 1x HAT every third day. On day 8 cells were trypsinized, counted and 1 × 10^6^ cells were seeded in medium containing 3 μg/mL puromycin (Sigma-Aldrich P8833). At the same time, 1 × 10^4^ cells were seeded in medium without puromycin as a seeding control. Cells were cultured for 7–10 days until colonies formed. Colonies were then counted and normalized.

#### TREX2 knockdown by shRNA in human cancer cell lines

Five × 10^6^ cells were mixed with PBS containing 10 μg mixture of three TREX2 shRNA plasmids, and electroporated for gene transduction. The conditions were: 230 mV, 960 μF for HCT 116 and SK-OV-3 cells; 200 V, 960 μF for CCRF-CEM cells. For attached cells (HCT 116, SK-OV-3), 1 × 10^4^ cells were plated in medium with 2 μg/mL puromycin on a 10 cm dish 24 hours after electroporation. After 7–10 days, 20 colonies were picked and expanded in medium containing puromycin. For CCRF-CEM cells, 100 μL cells from the selection step were seeded at a density of 10 cells/mL in a 96-well tissue culture plate, only wells containing 1 cell are considered monoclonal. Only cells in monoclonal wells were expanded and subjected to evaluation of TREX2 expression. Expanded cells were lysed and whole-cell lysate was subjected to Western blot for evaluation of TREX2 expression. TREX2 monoclonal antibody is from Santa Cruz (Catalog #sc-390890). HCT 116, CCRF-CEM, and SK-OV-3 cells were purchased from ATCC (CCL-247, CCL-119 and HTB-77, respectively).

Control shRNA plasmid-A was from Santa Cruz (Catalog #sc-108060).

TREX2 shRNA mixtures contain 3 different MISSION shRNAs from Sigma-Aldrich.

(Gene Symbol: TREX2, Gene ID: 11219, RefSeq ID: NM_080701, Species: human), TRC Number: TRCN0000413874, clone ID NM_080701.3-717s21c1: CCGGACAAGCTCACGCTGTGCATGTCTCGAGACATGCACAGCGTGAGCTTGTTTTTTTG, TRC Number: TRCN0000431678, clone ID NM_080701.3-897s21c1: CCGGATGGCTTTGATTATGATTTCCCTCGAGGGAAATCATAATCAAAGCCATTTTTTTG, TRC Number: TRCN0000426635, clone ID NM_080701.3-623s21c1: CCGGATTGCCGAGCTGTCCCTCTTTCTCGAGAAAGAGGGACAGCTCGGCAATTTTTTTG).

#### Construction of the *GFP(A)*_*10*_ polymerase slippage reporter

P-eGFP-N1 plasmid was obtained from Addgene (Catalog #6085-1). GFP coding sequence is amplified by PCR using the following custom primers from Sigma-Aldrich:

Forward 5’-TCACTCAGTCAGTAGACTCGAGCCGCCACC ATG GAA TTC AAA AAA AAA A GGA TCC GTGAGCAAGGGCGAGGAGCTGT-3’; Reverse 5’-TTCACGATGACCTGG GCGGCCGC TTACTTGTACAGCTCGTCCA-3’.

PCR product was gel extracted and cut with Xho1 and Not1 (37°C 4 hours). Plasmid was also cut with Xho1 and Not1 (37°C 4 hours). PCR fragments containing GFP(A)_10_ and plasmid were ligated by T4 DNA ligase (Roche 10 481 220 001) and ligation products were transduced into DH10β competent bacteria (Invitrogen 18-290-015) cells by electroporation. Bacteria were then plated on LB agar containing carbenicillin and cultured at 37°C.

#### Construction of the *Luciferase(A)*_*10*_ polymerase slippage reporter

Luciferase coding sequence was amplified using pCMV-Red Firefly Luc plasmid from Fisher Scientific (Catalog #16156) as the template plus the two custom primers from Sigma-Aldrich listed below that contain the out-of-frame ten adenines [(A)_10_]: Forward 5’ CACTCACTCAGTCAGTAGACCTCGAGGCCTTAGTGGCCGCCGCCACCAT GAAAAAAAAAAGAAAATATGGAAAACGACG 3’; Reverse 5’ TGCTGTTCACGATGACCTGG CTCGAG GCCACGCAGGCC TCA CATCTTGGCCACGGGTTTC 3’. PCR product was gel extracted using a QIAquick Gel Extraction Kit (Catalog #28706) and cut with Sfi1 from NEB (Catalog #R0123) at 37° C for 4 hours. Plasmid was also cut with Sfi1 at 37° C for 4 hours. T4 DNA ligase (Roche 10 481 220 001) was used to ligate the Luciferase (A)_10_ PCR product and the plasmid. Ligated product was transduced into DH10β competent bacteria cells (Invitrogen 18290-015) by electroporation. Bacteria were then plated on LB agar containing carbenicillin and cultured at 37°C. On the next day, 10 colonies were picked and sent for sequence to confirm (A)_10_ insertion.

#### *In vitro* luciferase assay

Luciferase activity is evaluated using Pierce^™^ Firefly Luciferase Glow Assay Kit (Catalog #16176). Briefly, cells were suspended at a concentration of 1 × 10^6^/mL in 1X DPBS buffer (Thermo Scientific BupH Modified Dulbecco’s PBS, Product No. 28374), 100 μL cell suspension was added to 100 μL of 1x Cell Lysis Buffer. The plate shook on a platform shaker at moderate speed for 15 minutes. Complete cell lysis was assessed using a light microscope. Bioluminescence signal was evaluated on luminometer. First, 10–20 μL/well of cell lysate was added to an opaque black, clear bottom 96-well plate. Then 50 μL of Working Solution was added to each well. Wait 10 minutes for signal stabilization and detect the light output at 30 second intervals. Six readings were recorded and averaged to achieve accurate results.

#### Knockout of RAD18

CHOPCHOP^30^ was used to design the *Rad18* gRNA plasmid. The *Rad18* exon1 oligo (CACCGAGGTCCTGGCCGAGCCGCGA) from Sigma-Aldrich was inserted into the pX330 vector^29^. For *msh2*^−/−^ ESCs, 5 × 10^6^ cells were co-transduced with 5 μg *MmRad18* gRNA and 5 μg neomycin resistant vector by electroporation as previously described^43^. Twenty-four hours after electroporation, cells were transferred to medium containing 6x G418 (Gibco 11811-023) for selection and 7–10 days later, 50 G418-resistant colonies were picked and transferred to 96-well plates to expand. Cells from each colony were then lysed and subjected to Western blot to evaluate RAD18 expression. The clone with no RAD18 expression was selected for further experimentation. The genomic DNA was extracted to amplify *RAD18* exon 1 by PCR as described^18^ to confirm mutation. Both RAD18 wild type and *rad18*−/− cells were then subjected to the *puro(A)*_*10*_ polymerase slippage assay as described above.

#### Dose response curve

The conditions for the dose response curve have been described^23^. Briefly, on Day 0, 2×10^3^ mouse ES cells were seeded onto a 24 well plate. On Day 1, the cells were treated with HU (20, 40, 60, 80 μM), APH (50, 100, 150, 200, 250 μM), and CPT (20, 40, 60, 80, 100, 120 μM). On Day 7, the cells were washed with PBS twice, trypsinized and counted. The experiment was performed three times.

#### DNA fiber analysis

The conditions for fiber analysis have been described^26^. Briefly, the cells were pulsed with 250 μM CldU for 20 minutes, treated with 0.5 mM HU for 1.5 hours, and pulsed with 25 μM IdU for 20 minutes. The cells were harvested and mixed with unlabeled cells (1:10). To prepare DNA fiber spreads, the cells were lysed with the spreading buffer (200 mM Tris-Cl, pH 7.4), 50 mM EDTA, 0.5% SDS) onto microscope slides and incubated for 2 minutes. The slides were tilted at 15° and the fibers were allowed to spread slowly down the slides. Then, the fibers were fixed in methanol/acetic acid (3:1) for 10 minutes and denatured in 2.5 M HCl for 1.25 hours. The fibers were rinsed with PBS and incubated in blocking buffer (1% BSA, 0.1% Tween20) for 30 minutes and incubated with rat *a*-BrdU (1:650) and mouse *a*-BrdU (1:650) in blocking buffer for 1 hour. Then, the fibers were fixed in 4% paraformaldehyde for 10 minutes and incubated with secondary antibodies (anti-rat AlexaFluor 555 and anti-mouse AlexaFluor 488, 1:500) in blocking buffer for 1.5 hours. DNA fibers were captured with Axio Imager A2 at 63x magnification (Zeiss) and analyzed using Zen 2.3pro software (Zeiss). At least 200 fibers were counted and analyzed.

### QUANTIFICATION AND STATISTICAL ANALYSIS

The statistical analysis was performed using Prism10 software (GraphPad). For cell survival assay, unpaired student T test were used. For DNA fiber assay, Fisher’s exact T test was used. For two-color FISH assay, Chi-square with Yates’ correction and Fisher’s exact test were used. Details regarding the number of replicates, corresponding p-values, and statistical tests are described in the figure legends.

## Supplementary Material

1

2

3

## Figures and Tables

**KEY RESOURCES TABLE:** 

REAGENT or RESOURCE	SOURCE	IDENTIFIER

Antibodies		

Mouse anti-TREX2 (E-4)	Santa Cruz	Cat# sc-390890
Rabbit anti-Rad18 (EPR8942)	Abcam	Cat# ab157463
Mouse anti-*β*-actin (C-4)	Santa Cruz	Cat# sc-47778, RRID: AB_626632
Goat anti-actin (C-11)	Santa Cruz	Cat# sc-1615, RRID: AB_630835
Rabbit IgG HRP linked whole ab	GE Healthcare	Cat# NA934 RRID: AB_772206
Mouse IgG HRP linked whole ab	GE Healthcare	Cat# NA931 RRID: AB_772210
Rat monoclonal anti-BrdU [BU1/75 (ICR1)]	AbD Serotec (Bio-Rad)	Cat# MCA2060T, RRID:AB_10015293
Mouse monoclonal anti-BrdU (B44)	BD Biosciences	Cat# 347580, RRID:AB_10015219
Goat anti-rat AlexaFluor555	Thermo Fisher	Cat# A-21434, RRID:AB_141733
Goat anti-mouse AlexaFluor488	Thermo Fisher	Cat# A-11017, RRID:AB_143160

Chemicals, peptides, and recombinant proteins		

Mitomycin C	Sigma-Aldrich	Cat# M4287
Meso-4,4-(2,3-butanediyl)-bis(2,6-piperazinedione)	ICN Biomedicals	
Etoposide	Sigma-Aldrich	Cat# E1383
Cisplatin	Sigma-Aldrich	Cat# P4394
5,6-Dichloro-1-*β*-D-ribofuranosylbenzimidazole	Calbiochem	Cat# 287891
Hydrogen peroxide	Sigma-Aldrich	Cat# 216763
N-ethyl-N-nitrosourea	Sigma-Aldrich	Cat# N8509
Methy methanesulphonate	Sigma-Aldrich	Cat# M4016
Hydroxyurea	Sigma-Aldrich	Cat# H8627
Camptothecin	Sigma-Aldrich	Cat# C9911
6-Thioguanine	Sigma-Aldrich	Cat# A4882
Puromycin	Sigma-Aldrich	Cat# P8833
50x HAT	Sigma-Aldrich	Cat# H0262
50x HT	Sigma-Aldrich	Cat# H0137
Aphidicolin	Sigma-Aldrich	Cat# A0781
Trypan blue	Gibco	Cat# 15250-061
G418	Gibco	Cat# 11811-023

Experimental models: Cell lines		

Mouse: AB1.1 Trex2^WT^	This paper	N/A
Mouse: AB1.1 Trex2^Null^	This paper	N/A
Mouse: AB1.1 MSH2^WT^ Trex2^WT^	This paper	N/A
Mouse: AB1.1 MSH2^WT^ Trex2^null^	This paper	N/A
Mouse: AB1.1 MSH2^WT^Trex2^H188A^	This paper	N/A
Mouse: AB1.1 MSH2^WT^Trex2^R167A^	This paper	N/A
Mouse: AB1.1 MSH2^null^ mTrex2^WT^	This paper	N/A
Mouse: AB1.1 MSH2^null^ Trex2^WT^	This paper	N/A
Mouse: AB1.1 MSH2^null^ Trex2^null^	This paper	N/A
Mouse: AB1.1 MSH2^null^ Trex2^H188A^	This paper	N/A
Mouse: AB1.1 MSH2^null^ Trex2^R167A^	This paper	N/A
Mouse: AB1.1 MSH2^null^ RAD18^WT^	This paper	N/A
Mouse: AB1.1 MSH2^null^ RAD18^null^	This paper	N/A
Human: HCT116 shCTRL	This paper	N/A
Human: HCT116 shTREX2	This paper	N/A
Human: SK-OV-3 shCTRL	This paper	N/A
Human: SK-OV-3 shTREX2	This paper	N/A
Human: CCRF-CEM shCTRL	This paper	N/A
Human: CCRF-CEM shTREX2	This paper	N/A

Oligonucleotides		

See Table S2 for oligonucleotides	N/A	N/A

Recombinant DNA		

px330-U6-Chimeric_BB-CBh-hSpCas9	Addgene	RRID: Addgene_42230
pPGKcrepA	*Kim* et al.^20^	N/A
pCAGGS-FLPe	*Kim* et al.^20^	N/A
CMKI-hTrex2 cDNA (WT)	*Chen* et al.^15^	N/A
CMKI-hTrex2 cDNA (Null)	*Chen* et al.^15^	N/A
CMKI-hTrex2 cDNA (H188A)	*Chen* et al.^15^	N/A
CMKI-hTrex2 cDNA (R167A)	*Chen* et al.^15^	N/A
Control shRNA plasmid A	Santa Cruz	Cat# sc-108060
P-eGFP-N1 plasmid	Addgene	Cat# 6085-1
pCMV-Red Firefly Luc plasmid	Fisher Scientific	Cat# 16156
Puro(A)_10_ reporter	This study	N/A
Puro(T)_11_ reporter	This study	N/A
Puro(T)_10_ reporter	This study	N/A
Puro(GT)_5_ reporter	This study	N/A
Puro(GT)_4_ reporter	This study	N/A
Puro(AC)_5_ reporter	This study	N/A
GFP(A)_10_ reporter	This study	N/A
Luciferase(A)_10_ reporter	This study	N/A

Software and algorithms		

Zen 2.3 pro	Zeiss	RRID: SCR_013672
Prism10	GraphPad	RRID: SCR_002798

Other		

Firefly Luciferase Glow Assay Kit	Pierce	Cat# 16176
QuickChange Multi Site-Directed Mutagenesis Kit	Stratagene	Cat# 200514

## Highlights

- The 3′–5′ exonuclease TREX2 causes spontaneous mutations in cells
- TREX2 causes mutations in cells exposed to a range of genotoxins
- TREX2 causes spontaneous mutations in MMR-deleted cells
- Deletion of TREX2 and MMR has a synthetic effect on DNA replication
