# Supplementary material for: TREX2 deficiency suppresses spontaneous and genotoxin-associated mutagenesis
Source: Cell Rep. Author manuscript; Available in PMC 2024 Feb 22. (PMC10883656; doi:10.1016/j.celrep.2023.113637)
Supplement: 1 [file NIHMS1961510-supplement-1.pdf]

Cell Reports, Volume *43*

Supplemental information

TREX2 deficiency suppresses spontaneous and genotoxin-associated mutagenesis

Teresa Marple, Mi Young Son, Xiaodong Cheng, Jun Ho Ko, Patrick Sung, and Paul Hasty

Supplemental figures

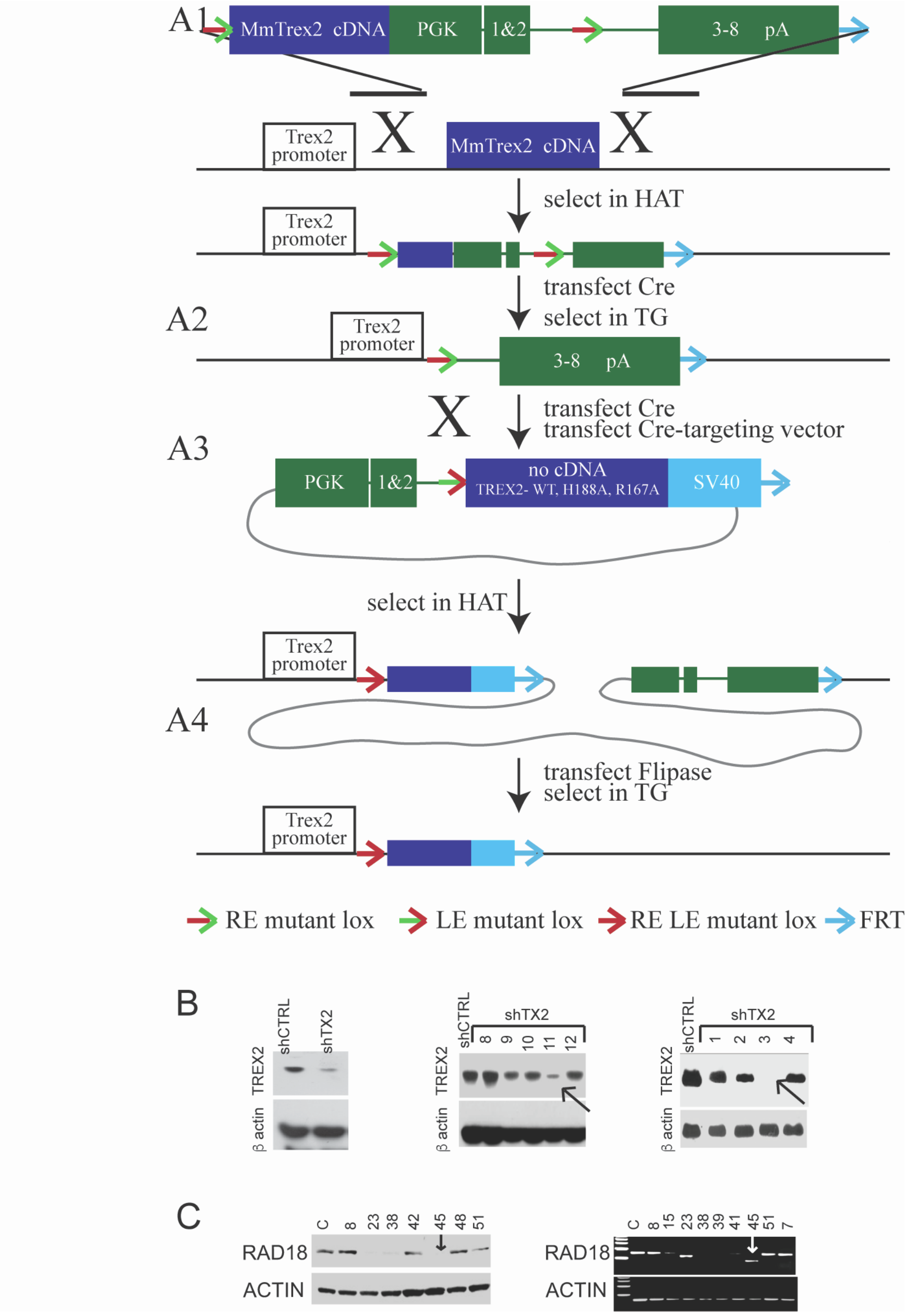

Figure S1. Generation of genetically altered cells. (**A1**) Knockout-knockin into *Trex2*. Replacement of mouse *Trex2* with a floxed mouse *Trex2-miniHPRT*[1,2]. A *loxP* flanks the 5' end and the intron of *miniHPRT*. An FRT (blue arrow) flanks the 3' end. Select in HAT. Screen with PCR. (**A2**) Generation of *TREX2*^{3mH} cells. The 5' half of *miniHPRT* was removed by transfection with Cre-recombinase followed

by selection in TG and screened for removal of 5’ *miniHPRT* by PCR. (**A3**) Knockin of Cre-mediated targeting vector with human *TREX2* cDNA (empty vector for null). Colonies selected in HAT, screen by PCR. (**A4**) Removal of plasmid backbone and *miniHPRT*. Cells were transfected with a *flippase* recombinase plasmid, selected in TG, screened by PCR. (**B**) Knockdown of TREX2 measured by Western analysis in HCT 116 cells (left panel), SK-OV-3 cells (middle panel) and CCRF-CEM (right panel) cells. Arrow points to clone used for transfection. (**C**) Knockout of RAD18 in cells measured by Western analysis (left panel) and PCR analysis (right panel). Clone #45 was used. This is a deletion of exon 1.

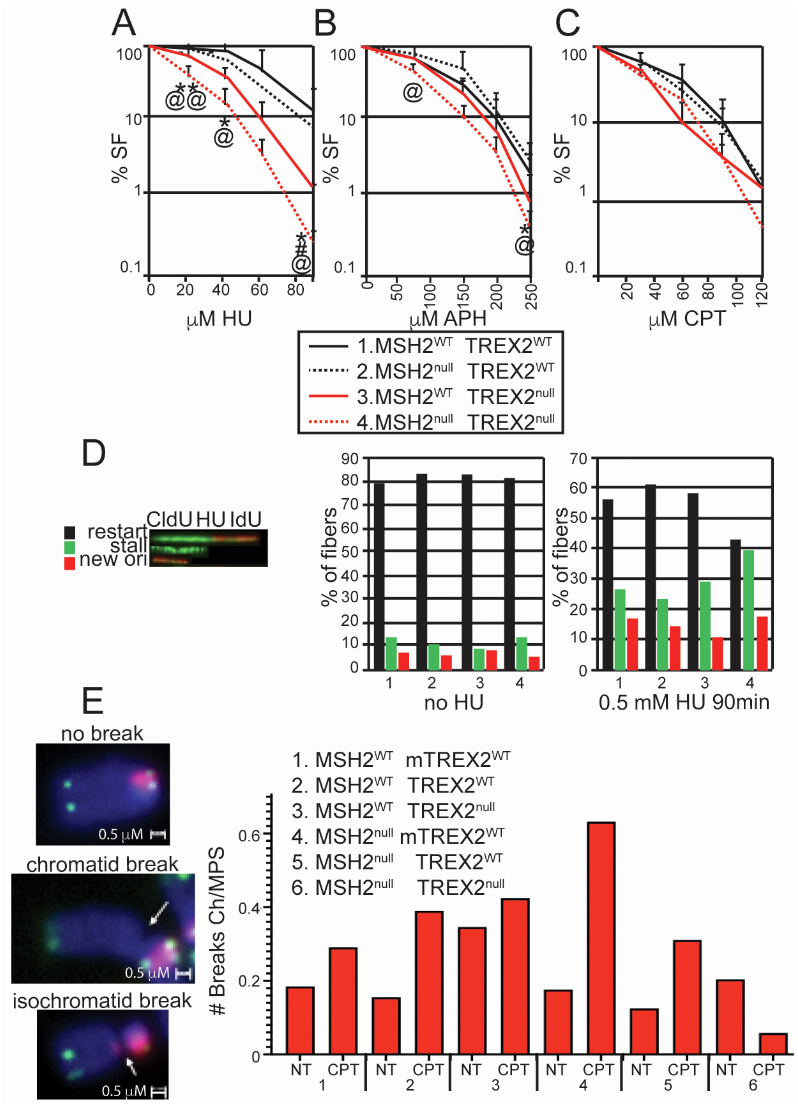

Figure S2. TREX2-deletion causes a synthetic phenotype in MSH2^{null} cells. Exposure to (**A**) HU, (**B**) APH, (**C**) CPT. Survival fraction, SF. Mean ± SD, n=3. Statistics: average of 3 replicates using the unpaired student T test: 2 vs 4: $p<0.05$ ∗, $p<0.01$ ∗∗. 1 vs 3: $p<0.05$, #. 1 vs 4 $p<0.05$, @; $p<0.01$, @@. (**D**) Fiber analysis. Left panel demonstrates fibers that show on going replication or RF restart, without or with HU, respectively (green-red shown as black), new origin (red) and RF stall (green). Middle panel represents the proportion of fibers from unexposed cells. Right panel represents the proportion of fibers from cells exposed to 0.5 mM HU for 1.5 hours. Total number of fibers observed without and with HU: 1 (1136, 415), 2 (764, 444), 3 (295, 865), 4 (423, 633). Statistics using the Fisher's exact T test. New origins for HU treated cells: 1 v 3: p=0.0096, 2 v 4: p=0.0452, 3 v 4, p=0.0003. Stalled RFs for HU treated cells: 1 v 4: $p<0.0045$, 2 v 4: $p<0.0002$, 3 v 4: $p<0.0159$. (**E**) Two-color Fluorescence in situ Hybridization (FISH) on metaphase spreads (MPSs) was used to screen for breaks: chromatid breaks (CBs) and isochromatid breaks (ICBs) with no treatment (NT) or treated with 60 nM CPT for 16 hours. MPSs were stained with a telomeric probe (green), a major satellite repeat probe (red) in the pericentromere and counterstained with DAPI (blue)[3]. Arrows point to break. At least 103 MPSs were analyzed and counted for each genotype. Statistics: a Chi-square with Yates' correction and Fisher's exact test were performed using Prism10. Scale bar: 0.5 µM

References

1. Kim, T.M., Ko, J.H., Hu, L., Kim, S.A., Bishop, A.J., Vijg, J., Montagna, C., and Hasty, P. (2012). RAD51 mutants cause replication defects and chromosomal instability. Mol Cell Biol *32*, 3663-3680. 10.1128/MCB.00406-12.
2. Holcomb, V.B., Kim, T.M., Dumitrache, L.C., Ma, S.M., Chen, M.J., and Hasty, P. (2007). HPRT minigene generates chimeric transcripts as a by-product of gene targeting. Genesis *45*, 275-281. 10.1002/dvg.20300.
3. Guenatri, M., Bailly, D., Maison, C., and Almouzni, G. (2004). Mouse centric and pericentric satellite repeats form distinct functional heterochromatin. J Cell Biol *166*, 493-505. 10.1083/jcb.200403109.
